# A Surveillance Study of Culturable and Antimicrobial-Resistant Bacteria in Two Urban WWTPs in Northern Spain

**DOI:** 10.3390/antibiotics13100955

**Published:** 2024-10-11

**Authors:** Mario Sergio Pino-Hurtado, Rosa Fernández-Fernández, Allelen Campaña-Burguet, Carmen González-Azcona, Carmen Lozano, Myriam Zarazaga, Carmen Torres

**Affiliations:** Area of Biochemistry and Molecular Biology, OneHealth-UR Research Group, University of La Rioja, 26006 Logroño, Spain; mario-sergio.pino@unirioja.es (M.S.P.-H.); allelen.campana@unirioja.es (A.C.-B.);

**Keywords:** antimicrobial resistant, bacterial diversity, CR-E, ESBL, ESKAPE, VRE, WWTPs

## Abstract

Background/Objectives: Wastewater treatment plants (WWTPs) are hotspots for the spread of antimicrobial resistance into the environment. This study aimed to estimate the proportion of clinically relevant antimicrobial-resistant bacteria in two Spanish urban WWTPs, located in the region of La Rioja (Spain); Methods: Ninety-four samples (48 water/46 sludge) were collected and streaked on ten different selective media, in order to recover the culturable bacterial diversity with relevant resistance phenotypes: Extended-Spectrum β-Lactamase-producing *Escherichia coli*/*Klebsiella pneumoniae* (ESBL-Ec/Kp), Carbapenem-resistant *Enterobacteriaceae* (CR-E), Methicillin-resistant *Staphylococcus aureus* (MRSA), and Vancomycin-resistant *Enterococcus faecium*/*faecalis* (VR-*E. faecium*/*faecalis*). Isolates were identified by MALDI-TOF and were tested for antimicrobial susceptibility using the disk diffusion method. The confirmation of ESBL production was performed by the double-disk test; Results: A total of 914 isolates were recovered (31 genera and 90 species). Isolates with clinically relevant resistance phenotypes such as ESBL-Ec/Kp and CR-E were recovered in the effluent (0.4 × 10^0^–4.8 × 10^1^ CFU/mL) and organic amendment samples (1.0–10^1^–6.0 × 10^2^ CFU/mL), which are discharged to surface waters/agricultural fields. We reported the presence of VR-*E. faecium* in non-treated sludge and in the digested sludge samples (1.3 × 10^1^–1 × 10^3^ CFU/mL). MRSA was also recovered, but only in low abundance in the effluent (0.2 × 10^1^ CFU/mL); Conclusions: This study highlights the need for improved wastewater technologies and stricter regulations on the use of amendment sludge in agriculture. In addition, regular monitoring and surveillance of WWTPs are critical for early detection and the mitigation of risks associated with the spread of antimicrobial resistance.

## 1. Introduction

Antimicrobial resistance (AMR) is a threat to the health of humans, animals, and the environment [1,2]. The indiscriminate use of antibiotics in the medical and agricultural sectors is the primary cause of the rapid appearance of this “silent pandemic”. Indeed, the World Health Organization (WHO) included it as one of the top ten threats to global health in 2019 [3].

Wastewater treatment plants (WWTPs) are very important for environmental protection and public health, but these systems are also a hotspot for the release and dissemination of AMR into environment [4]. The WWTPs receive discharges from different sources and are unable to completely remove traces of antibiotics, antimicrobial-resistant bacteria (ARB), and antimicrobial resistance genes (ARGs) from wastewater, making them a convergence point for these contaminants that are later released into the surface waters [5,6]. During conventional mechanical–biological treatment, due to the high microbial density, bacteria are in constant interaction with each other, as well as with traces of antibiotics and heavy metals at subinhibitory concentrations that promote the possible co-selection of ARB [5]. Therefore, it is hypothesized that WWTP effluent is one of the major anthropogenic sources of ARB and ARGs in the environment [7,8]. Sewage sludge is also an important reservoir of these biological contaminants of emerging concern, but few studies have examined the impact of sludge application to agricultural fields on AMR [9,10].

The ESKAPE group includes the most challenging opportunistic pathogens for multidrug resistance (MDR), an acronym reflecting their ability to “escape” antibiotics. The pathogens listed are Vancomycin-resistant *Enterococcus faecium* (VR-*E. faecium*), Methicillin-resistant *Staphylococcus aureus* (MRSA), or Carbapenem-resistant-*Klebsiella pneumoniae*; -*Acinetobacter baumannii*, -*Pseudomonas aeruginosa*, and -*Enterobacter* spp. [1].

Methicillin-resistant *S. aureus* is an opportunistic pathogen and a frequent cause of nosocomial infections. This pathogen is usually not detected in the environment, especially in wastewater [11]. Meanwhile, VR-*E. faecium*/*faecalis* are well known pathogens of hospital-acquired infections, particularly relevant in cases of bacteremia or endocarditis [12]. Due to the presence of enterococci in the intestinal tract of humans and animals, it is used as an indicator of fecal contamination in water [13]. However, there are fewer studies on the effect of wastewater treatment on Gram-positive pathogens or evidence of their spread through effluent compared to Gram-negative pathogens [14,15,16,17]. Members of the *Enterobacteriaceae* family are of most concern in wastewater and, to a higher degree, Extended-Spectrum β-Lactamase-producing *Escherichia coli*/*K. pneumoniae* (ESBL-*Ec*/*Kp*), and Carbapenem-resistant *Enterobacteriaceae* [18,19,20,21,22,23,24].

It is very important to determine the proportion of ARB in the bacterial diversity present in WWTPs (influent, effluent, and organic amendment) to determine the contribution of these facilities to the spread of AMR and the potential public health impact generated. With this information, it would be possible to implement effective actions to mitigate this problem, a field in which gaps still exist. Therefore, this study aimed to estimate the abundance and proportional decrease of several clinically relevant pathogenic bacteria during the wastewater and sewage sludge treatment processes of two urban WWTPs in northern Spain.

## 2. Results

### 2.1. Culturable Bacterial Diversity

#### 2.1.1. Bacterial Collection Recovered from Both WWTPs

**Figure 1:**
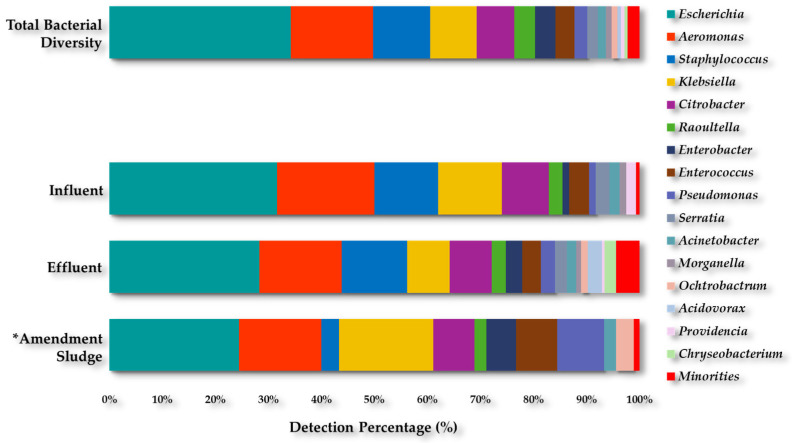
Diversity of the bacterial collection recovered from both WWTPs. * Amendment sludge samples (including Digested sludge and Organic amendment). Minorities: *Kluyvera*; *Leclercia*; *Stenotrophonas*; *Brevundimonas*; *Hafnia*; *Agrobacterium*; *Comamonas*; *Erysipelothrix*; *Exiguobacterium*; *Empedobacter*; *Lactobacillus*; *Micrococcus*; *Myroides*; *Rahnella*; and *Streptococcus*. Only *Hafnia* was detected as a minority genus in the influent samples and *Empedobacter* in the organic amendment.

A total collection of 914 isolates were recovered from all the media during the sampling period in both WWTPs (Gram-negative 85% and Gram-positive 15%), corresponding to thirty-one genera and ninety species. The genera recovered were as follows: *Escherichia* (34.2%); *Aeromonas* (15.5%); *Staphylococcus* (10.7%); *Klebsiella* (8.8%); *Citrobacter* (7.1%); *Raoultella* (3.9%); *Enterobacter* (3.8%); *Enterococcus* (3.6%); *Pseudomonas* (2.4%); *Serratia* (2.0%); *Acinetobacter* (1.5%); *Morganella* (1.1%); *Ochtrobactrum* (1.1%); *Acidovorax* (0.7%); *Providencia* (0.7%); *Chryseobacterium* (0.5%); among other minority genera (2.3%) (Figure 1).

One hundred fifty-eight isolates (n = 158) were recovered from the Influent (I) samples, corresponding to fourteen genera and thirty-seven species. Two hundred twenty-eight isolates (n = 128) were recovered from the Effluent (E) samples, corresponding to twenty-five genera and sixty species. Meanwhile, ninety bacterial isolates (n = 90) were recovered from the Amendment sludge samples (including Digested sludge and Organic amendment), corresponding to twelve genera and thirty-four species.

#### 2.1.2. Diversity of *Aeromonas* spp. and *Pseudomonas* spp.

**Figure 2:**
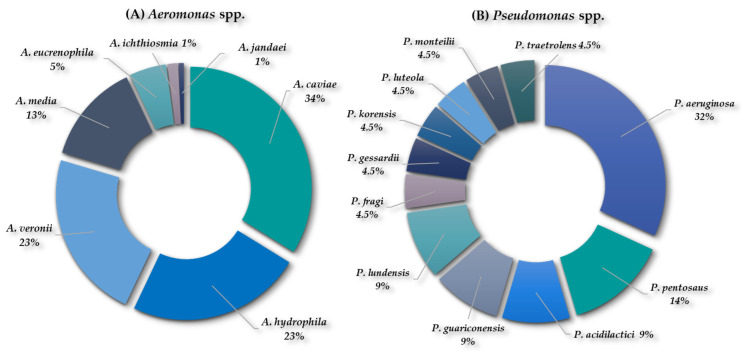
Percentage of detection of (**A**) *Aeromonas* spp. and (**B**) *Pseudomonas* spp. recovered from both WWTPs.

One hundred forty-two isolates (n = 142) were identified as *Aeromonas* spp., corresponding to seven species, as follows: *A. caviae* (34.6%); *A. hydrophila* (25%); *A. veronii* (21.2%); *A. media* (10.6%); *A. eucrenophila* (5.8%); *A. ichthiosmia* (1.9%); and *A. jandaei* (1%) (Figure 2A).

In addition, twenty-two isolates were identified as *Pseudomonas* spp. (n = 22), corresponding to eleven species, as follows: *P. aeruginosa* (32%); *P. pentosaus* (14%); *P. acidilactici* (9%); *P. guariconensis* (9%); *P. lundensis* (9%); *P. fragi* (4.5%); *P. gessardii* (4.5%); *P. koreensis* (4.5%); *P. luteola* (4.5%); *P. monteilii* (4.5%); and *P. taetrolens* (4.5%) (Figure 2B).

#### 2.1.3. Diversity of Species of *Staphylococcus* spp. and *Enterococcus* spp.

**Figure 3:**
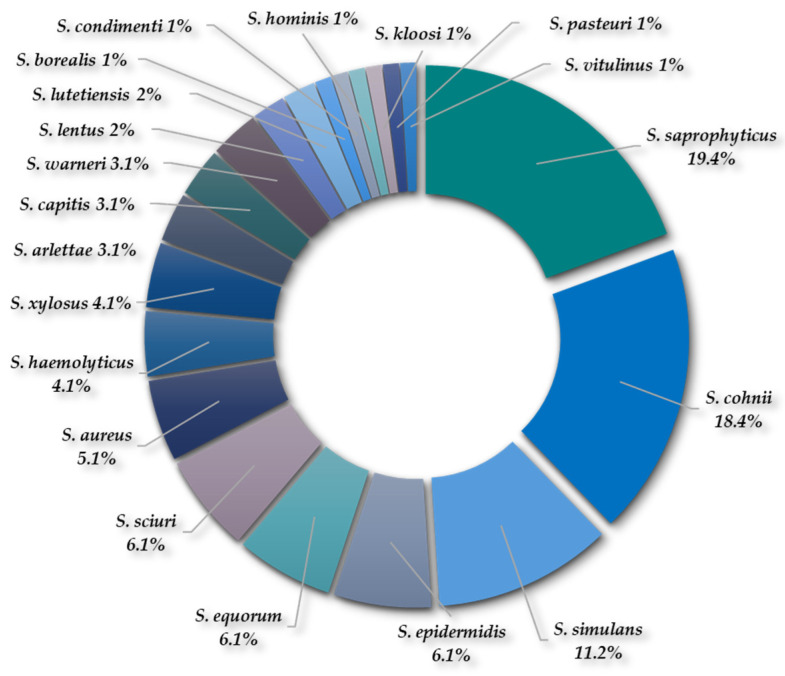
Percentage of detection of *Staphylococcus* spp. recovered from both WWTPs.

Ninety-eight isolates (n = 98) were recovered from the Mannitol Salt Agar plates and were identified as *Staphylococcus* spp., most of them Coagulase-negative staphylococci (CoNS). Twenty staphylococcal species were detected, as follows: *S. saprophyticus* (19.4%); *S. cohnii* (18.4%); *S. simulans* (11.2%); *S. epidermidis* (6.1%); *S. equorum* (6.1%); *S. sciuri* (6.1%); *S. aureus* (5.1%); *S. haemolyticus* (4.1%); *S. xylosus* (4.1%); *S. arlettae* (3.1%); *S. capitis* (3.1%); *S. warneri* (3.1%); *S. lentus* (2%); *S. lutetiensis* (2%); *S. borealis* (1%); *S. condimenti* (1%); *S. hominis* (1%); *S. kloosi* (1%); *S. pasteuri* (1%); and *S. vitulinus* (1%) (Figure 3). It is interesting to note that *S. sciuri*, *S. lentus* and *S. vitulinus* are now reclassified into the genus *Mammaliicoccus*.

**Figure 4:**
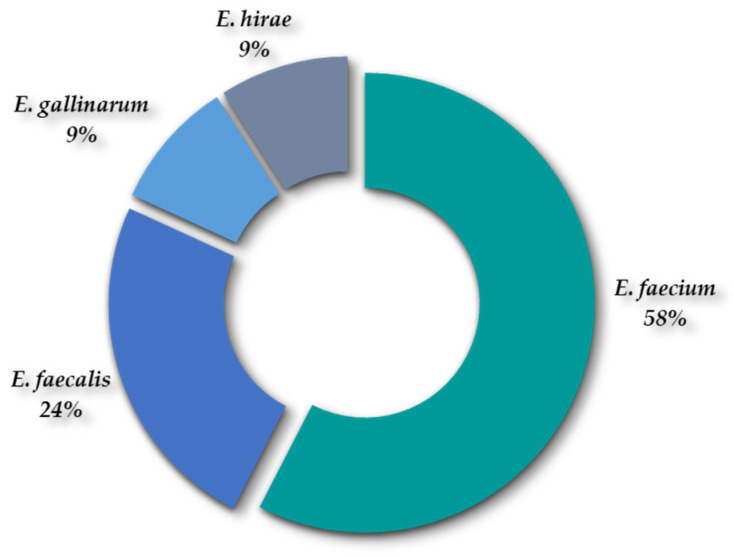
Percentage of detection of *Enterococcus* spp. recovered from both WWTPs.

Thirty-three isolates (n = 33) were recovered from the SB Agar plates (non-supplemented and supplemented with 4 µg/mL of vancomycin) and were identified as *Enterococcus* spp., corresponding to four species as follows: *E. faecium* (58%); *E. faecalis* (24%); *E. hirae* (9%); and *E. gallinarum* (9%) (Figure 4).

### 2.2. Detection of Clinically Relevant Antimicrobial-Resistant Bacteria

#### 2.2.1. Cefotaxime-Resistant *Enterobacteriaceae* (CTX^R^-E)

MacConkey agar plates non-supplemented and supplemented with 2 µg/mL of cefotaxime were used for the isolation of *Enterobacteriaceae* and CTX^R^-*Enterobacteriaceae*. A total of 204 isolates were recovered, corresponding to five genera and fourteen species, and were as follows: *Escherichia coli* (60.8%); *Klebsiella oxytoca* (9.8%); *Citrobacter freundii* (8.8%); *Klebsiella pneumoniae* (5.4%); *Raoultella ornithinolytica* (4.9%); *Citrobacter gillenii* (2%); *Enterobacter absuriae* (2%); *Enterobacter cloacae* (2%); *Citrobacter braakii* (1%); *Enterobacter kobei* (1%); *Klebsiella variicola* (1%); *Enterobacter bugandensis* (1%); *Klebsiella aerogenes* (1%); and *Raoultella planticola* (1%). 

**Figure 5:**
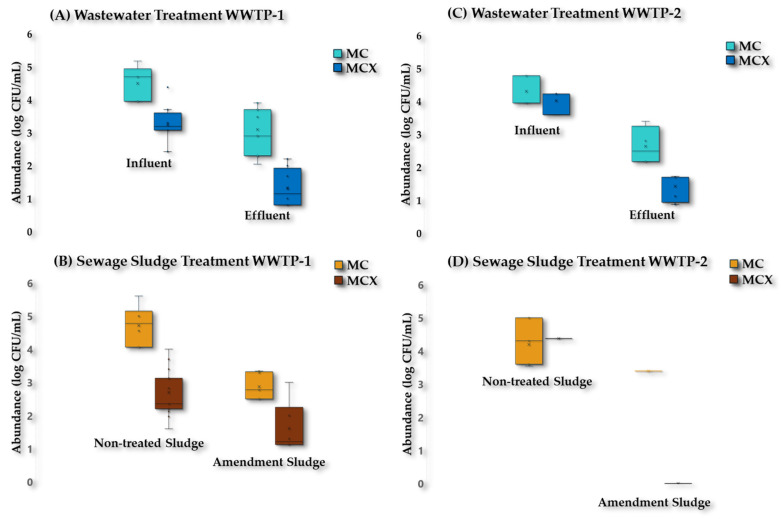
Abundance of the total *Enterobacteriaceae* and CTX^R^-E recovered on MC Agar plates non-supplemented (MC) and supplemented with CTX (MCX), respectively (log CFU/mL). Amendment sludge samples (including Digested sludge and Organic amendment).

A total of 51.5% of these isolates were recovered on plates supplemented with 2 µg/mL of cefotaxime (n = 105). The ratio of abundance of CTX^R^-E to the total *Enterobacteriaceae* recovered on MC Agar plates is shown in Figure 5.

During wastewater treatment (Figure 5A,C), there was a decrease of 1–2 log in the total abundance of *Enterobacteriaceae* from both WWTPs. The abundance of CTX^R^-E in the Influent (I) of WWTP-1 was in the range of 2.6 × 10^2^–2.4 × 10^4^ CFU/mL, and a decrease of 1–2 log units in the abundance of CTX^R^-E was observed in the Effluent (E). Meanwhile, in WWTP-2, a complete elimination was also not achieved, since CTX^R^-E were also recovered in the Effluent (E) samples (7.5 × 10^0^–1.3 × 10^1^ CFU/mL). From the total *Enterobacteriaceae* recovered in the effluent samples, 40% were CTX^R^-E.

During the sewage sludge treatment (Figure 5B,D), there was also a decrease of 1–2 log in the total abundance of *Enterobacteriaceae*. The abundance of CTX^R^-E decreased during sewage sludge treatment in WWTP-1, although these ARB were not completely eliminated. In this sense, it started at a concentration of 4.0 × 10^1^–1.0 × 10^4^ CFU/mL in non-treated sludge samples (Primary, Secondary, and Mixed sludge), and a decrease of 1–2 log units was observed in the Amendment sludge samples (including Digested sludge and Organic amendment), ranging from 1.3 × 10^1^ to 1.0 × 10^3^ CFU/mL. A total of 33.3% out of the total *Enterobacteriaceae* recovered in the amendment sludge of samples WWTP-1 was CTX^R^-E. Meanwhile, in WWTP-2, CTX^R^-E were only recovered in non-treated sludge samples (2.3 × 10^4^ CFU/mL).

#### 2.2.2. ESBL-Producing *E. coli*/*K. pneumoniae* (ESBL-Ec/Kp) Isolates

Chromogenic CHROMID^®^ ESBL Agar plates, and MCX Agar plates supplemented with 2 µg/mL cefotaxime were used for ESBL-Ec/Kp recovery. A total of one hundred and seventy-nine ESBL-Ec/Kp isolates (n = 179) were recovered from both WWTPs during the sampling period: *E. coli* (92.7%) and *K. pneumoniae* (7.3%). 

**Figure 6:**
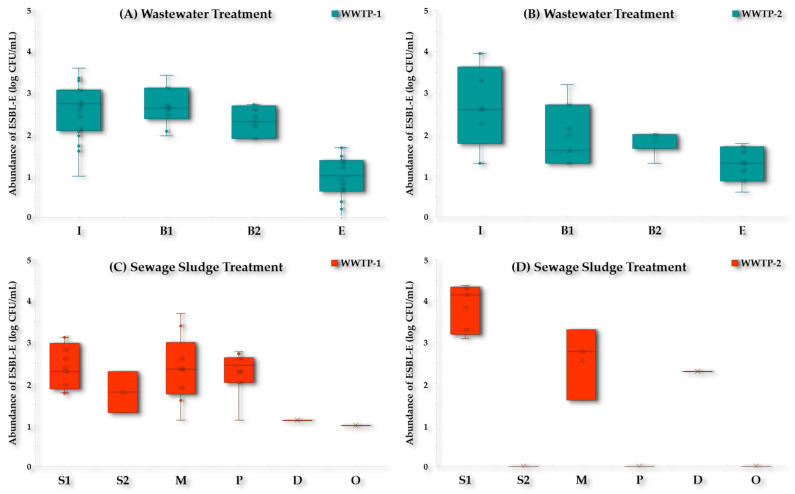
Abundance of ESBL-producing *E. coli*/*K. pneumoniae* (log CFU/mL) in both WWTPs during (**A**,**B**) wastewater treatment and (**C**,**D**) sewage sludge treatment. Abbreviations**:** Influent (I); Bioreactor input (B_1_); Bioreactor output (B_2_); Effluent (E); Primary sludge (S_1_); Secondary sludge (S_2_); Mixed sludge (M); Pasteurized sludge (P); Digested sludge (D); and Organic amendment (O).

There was a decrease in the abundance of ESBL-Ec/Kp in both WWTPs during wastewater treatment (Figure 6). In this sense, the counts of ESBL-Ec/Kp detected in the Influent (I) of WWTP-1 were in the range of 4.0 × 10^1^–4.0 × 10^3^ CFU/mL and they decreased by 1–3 log units to reach 0.4 × 10^0^–4.8 × 10^1^ CFU/mL in the Effluent (E) samples (Figure 6A). In relation to WWTP-2, the abundance of ESBL-Ec/Kp in the Influent (I) were 2.0 × 10^1^–9.0 × 10^3^ CFU/mL and also decreased 1–3 logs to 4.0 × 10^0^–5.0 × 10^1^ CFU/mL in the Effluent (E) samples (Figure 6B).

The abundance of ESBL-Ec/Kp during sewage sludge treatment in WWTP-1 ranged from 1.3 × 10^1^ to 2.5 × 10^3^ CFU/mL in the non-treated sludge samples (including Primary, Secondary, and Mixed sludge) to reach 1.0 × 10^1^ CFU/mL in the Organic amendment (O) samples (Figure 6C). In contrast, in WWTP-2, no growth of ESBL-Ec/Kp was detected in the Organic amendment (O) samples, although it was detected in the Digested sludge (D) samples with values of 2.0 × 10^2^ CFU/mL (Figure 6D).

#### 2.2.3. Carbapenem-Resistant *Enterobacteriaceae* (CR-E)

Chromogenic Brilliance^®^ CRE Agar plates were used for the recovery of fifty-one CR-E isolates (n = 51), representing six genera and thirteen species, as follows: *E. coli* (25%); *Citrobacter freundii* (24%); *K. pneumoniae* (12%); *Klebsiella oxytoca* (8%); *Enterobacter kobei* (8%); *Enterobacter absuriae* (4%); *Enterobacter bugandensis* (4%); *Enterobacter cloacae* (4%); *Raoultella ornithinolytica* (4%); *Citrobacter amalonaticus* (2%); *Citrobacter braakii* (2%); *Citrobacter farmeri* (2%); and *Klebsiella variicola* (2%).

**Figure 7:**
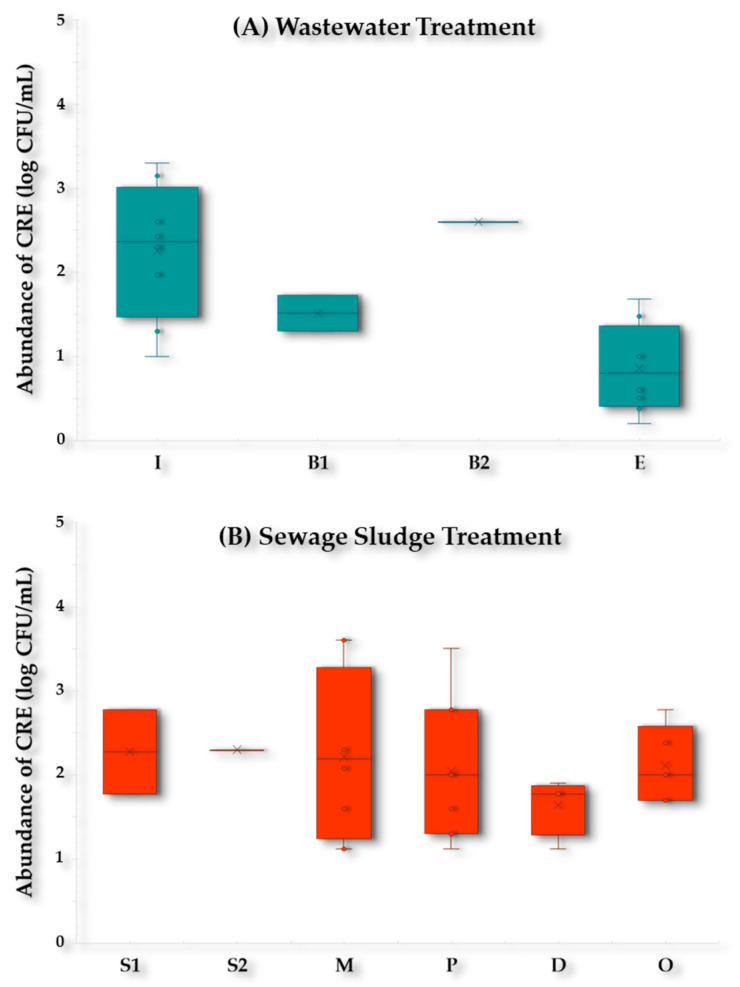
Abundance of Carbapenem-resistant *Enterobacteriaceae* (log CFU/mL) in WWTP-1 during (**A**) wastewater treatment and (**B**) sewage sludge treatment. Abbreviations: Influent (I); Bioreactor input (B1); Bioreactor output (B2); Effluent (E); Primary sludge (S1); Secondary sludge (S2); Mixed sludge (M); Pasteurized sludge (P); Digested sludge (D); and Organic amendment (O).

During wastewater treatment, the abundance of CR-E in WWTP-1 decreased by 1–3 logs, with concentrations of 1.0 × 10^1^–2 × 10^3^ CFU/mL in the Influent samples (I), to reach 1.6 × 10^0^–4.8 × 10^1^ CFU/mL in the Effluent (E) samples (Figure 7A). The abundance of CR-E during sewage sludge treatment in WWTP-1 ranged from 1.3 × 10^1^ to 4.0 × 10^3^ CFU/mL in the non-treated sludge samples (Primary sludge, Secondary sludge, and Mixed sludge samples) to reach abundances of 6.0 × 10^2^ CFU/mL in the Organic amendment (O) samples (Figure 7B).

Imipenem-resistant *R. ornithinolytica* isolates (IPM^R^-*R. ornithinolytica*) were detected in the Mixed sludge (M) (2.0 × 10^2^ CFU/mL) and Pasteurized sludge (P) samples (3.2 × 10^3^ CFU/mL) from WWTP-1. Meanwhile, only IPM^R^-*C. freundii* was detected in the Effluent (E) samples from WWTP-2 (3.0 × 10^1^ CFU/mL) and no CR-E were found in the sludge samples from this WWTP during the sampling period.

#### 2.2.4. *Enterobacteriaceae* Isolates Recovered from COLR Agar Plates

Sixty-five *Enterobacteriaceae* isolates were recovered from Chromogenic CHROMID^®^ COLR Agar plates, representing four genera and six species, as follows: *E. coli* (58%); *R. ornithinolytica* (17%); *K. pneumoniae* (12%); *K. oxytoca* (9%); *E. absuriae* (2%); and *K. variicola* (2%). These isolates will be further characterized in the future.

#### 2.2.5. Methicillin-Resistant *Staphylococcus aureus* and Vancomycin-Resistant *Enterococcus faecium*/*faecalis*

Four *S. aureus* isolates (n = 4) were recovered during the sampling period, all in the effluent (E) samples from WWTP-1 at concentrations of 1.3 × 10^1^–1.0 × 10^3^ CFU/mL. One of the four staphylococcal isolates was MRSA, detected at a concentration of 4 × 10^1^ CFU/mL.

Slanetz–Bartley Agar plates supplemented with 4 µg/mL of vancomycin were used for the isolation of VR-*E. faecium*/*faecalis*. Two MDR-VR-*E. faecium* isolates (n = 2) were recovered from the mixed sludge and pasteurized sludge samples at concentrations of 1.3 × 10^1^–1.0 × 10^3^ CFU/mL. However, no VRE were detected in the effluent or in the organic amendment samples.

### 2.3. Microbiological Impact of the WWTP-2 Collectors

**Figure 8:**
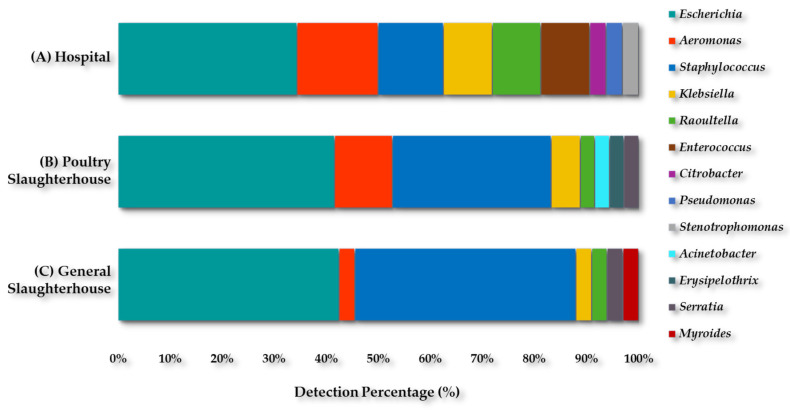
Diversity of culturable bacterial genera recovered from the WWTP-2 Collectors.

Three collectors discharging into WWTP-2 [Hospital (H), Poultry slaughterhouse (Ps), and General slaughterhouse (Gs)] were analyzed. A total of thirty-two isolates were recovered from the Hospital effluent (H) samples (Gram-negative 75% and Gram-positive 25%), representing nine genera and fourteen species, as follows: *Escherichia* (34.4%); *Aeromonas* (15.6%); *Staphylococcus* (12.5%); *Klebsiella* (9.4%); *Raoultella* (9.4%); *Enterococcus* (9.4%); *Citrobacter* (3.1%); *Pseudomonas* (3.1%); and *Stenotrophomonas* (3.1%) (Figure 8A).

Thirty-six isolates were recovered from the Poultry slaughterhouse effluent (Ps) samples (Gram-negative 69% and Gram-positive 31%), corresponding to eight genera and fifteen species, and were as follows: *Escherichia* (41.7%); *Staphylococcus* (30.6%); *Aeromonas* (11.1%); *Klebsiella* (5.6%); *Acinetobacter* (2.8%); *Erysipelothrix* (2.8%); *Raoultella* (2.8%); and *Serratia* (2.8%) (Figure 8B).

Meanwhile, a total of thirty-three isolates were recovered from the General slaughterhouse effluent (Gs) samples (Gram-negative 55% and Gram-positive 42%), corresponding to seven genera and eleven species and were as follows: *Escherichia* (42.4%); *Staphylococcus* (42.4%); *Aeromonas* (3.0%); *Klebsiella* (3.0%); *Myroides* (3.0%); *Raoultella* (3.0%); and *Serratia* (3.0%) (Figure 8C).

Twelve ESBL-Ec/Kp isolates were recovered from the Hospital effluent (H) samples (n = 12) in a concentration of 1.0 × 10^1^–4.0 × 10^3^ CFU/mL, *E. coli* (83%) and *K. pneumoniae* (17%). In addition, CR-*E. coli* was recovered in a concentration of 2.0 × 10^2^ CFU/mL.

Ten ESBL-Ec/Kp isolates were recovered from the Poultry slaughterhouse effluent (Ps) samples (n = 10) in a concentration of 4.7 × 10^0^–2.5 × 10^2^ CFU/mL, *E. coli* (90%) and *K. pneumoniae* (10%). Moreover, CR-*E. coli* was recovered in a concentration of 2.0 × 10^1^ CFU/mL.

Meanwhile, seven ESBL-*E. coli* isolates were recovered from the General slaughterhouse effluent (Gs) samples in a concentration of 1.2 × 10^1^–8.0 × 10^2^ CFU/mL. However, ESBL-*K. pneumoniae* or CR-E were not detected in this collector.

## 3. Materials and Methods

### 3.1. Criteria for the Selection of WWTP Sampling

We selected the two biggest WWTPs among those located in the region of La Rioja (Spain) (WWTP-1 and WWTP-2). These plants receive discharges from different anthropogenic sources (hospitals, rural or urban human domestic activities, and slaughterhouses).

#### 3.1.1. Characteristics of WWTP-1

**Figure 9:**
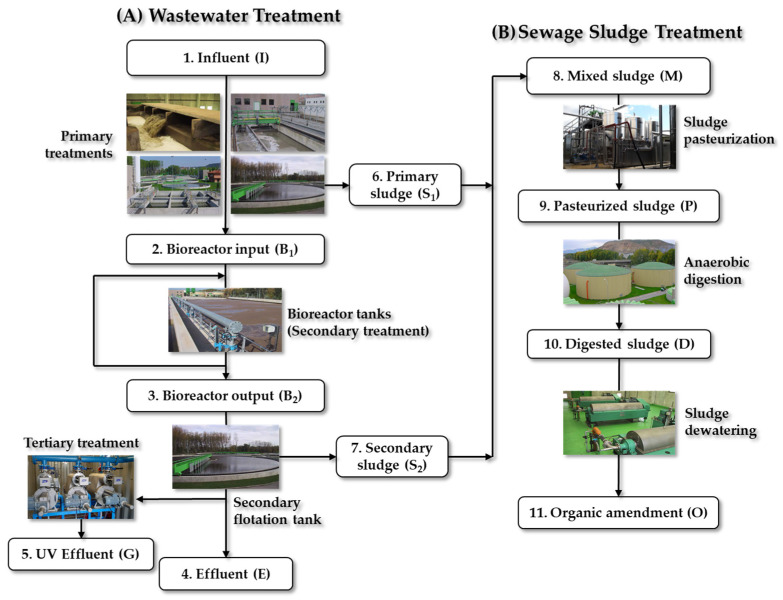
Sampling points established in WWTP-1. (**A**) Wastewater treatment and (**B**) sewage sludge treatment. https://larioja.org/consorcio-aguas/es/depuracion/instalaciones/depuradoras-servicio/d-r-logrono-bajo-iregua (accessed on 15 May 2024).

WWTP-1 was designed to treat the discharges of a population equivalent to 466,560 inhabitants and focuses on the elimination of up to 89% of nitrogen using half-load activated sludge technology. The WWTP-1 receives discharges from rural and urban human domestic activities and hospitals (but not from any slaughterhouses), although only the general collector discharging to this WWTP was analyzed and not the specific collectors. Figure 9 shows the workflow of WWTP-1. The effluent is discharged into the Ebro River. A portion of the treated water undergoes additional ultraviolet (UV) disinfection and reuse internally (tertiary treatment) (Figure 9A).

The sewage sludge produced is first pasteurized, subjected to an anaerobic digestion process, then dehydrated and finally reused as an organic amendment in agriculture (Figure 9B).

#### 3.1.2. Characteristics of WWTP-2

**Figure 10:**
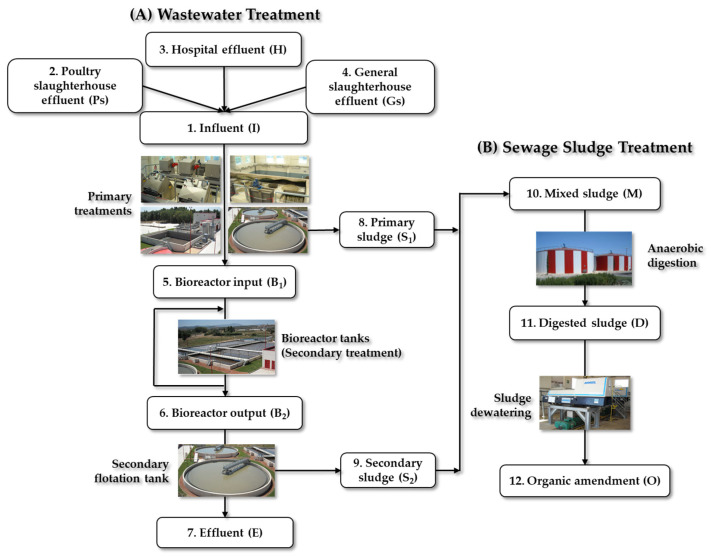
Sampling points established in WWTP-2. (**A**) Wastewater treatment and (**B**) sewage sludge treatment. https://larioja.org/consorcio-aguas/es/depuracion/instalaciones/depuradoras-servicio/d-r-calahorra (accessed on 15 May 2024).

WWTP-2 was designed to treat the discharges of a population equivalent to 143,000 inhabitants and focuses on the elimination of nitrogen and phosphorus using activated sludge technology in the anoxic-anaerobic-oxic (A_2_O) variant. The workflow of WWTP-2 is shown in Figure 10. WWTP-2 receives discharges from rural and urban human domestic activities, one hospital, and slaughterhouses. The WWTP effluent is discharged to the Cidacos River (Figure 10A).

The sewage sludge produced is also subjected to an anaerobic digestion process, dehydrated, and reused as an organic amendment (Figure 10B).

Three wastewater collectors discharging to this WWTP were selected for the microbiological impact analysis: Poultry slaughterhouse, Hospital, and General slaughterhouse. The term “General slaughterhouse” is used in reference to the slaughter of cattle, sheep, and pigs.

### 3.2. Samples Collection

In this study, a total of ninety-four samples (forty-eight water/forty-six sludge) were collected at different steps of wastewater and sludge treatment from the two analyzed WWTPs during the sampling period from January-2022 to December-2023.

Eleven sampling points were selected in WWTP-1 (five water/six sludge) and thirteen sampling points in WWTP-2 (seven water/six sludge). Wastewater and sludge samples were collected in sterile Pyrex bottles (1 L) and Falcon tubes (50 mL), respectively. All samples were kept cold and transported immediately to the laboratory for further analysis [4].

### 3.3. Samples Processing

Once the samples arrived at the laboratory, serial decimal dilutions from 10^0^ to 10^−4^ were prepared in MilliQ water (Merck, Barcelona, Spain). The volume/dilution inoculated depended on the characteristics of the culture media and the origin of each sample.

A volume of 10 µL (10^−3^ and 10^−4^ dilutions) of each sample (except those indicated below) was inoculated on Blood Agar (BA) plates, which is a nutrient-rich medium. Meanwhile, 50 µL of each sample was inoculated for the other culture media: chromogenic/supplemented media (10^0^ and 10^−1^ dilutions) and non-chromogenic/non-supplemented media (10^−1^ and 10^−2^ dilutions). The WWTPs effluent, slaughterhouse effluent, and organic amendment samples were processed differently, due to their characteristics.

The Effluent (E), Poultry slaughterhouse (Ps), and General slaughterhouse (Gs) samples (100–150 mL) were previously filtered through cellulose acetate membranes, pore size 0.22 µm (Sartorius, Göttingen, Germany). These membranes were then resuspended in Brain Heart Infusion Broth (Condalab, Madrid, Spain) and 100 µL of non-diluted samples was inoculated into the different culture media. The additional filtration steps were performed to concentrate the Effluent (E) samples, and the volume filtered to the point of membrane clogging was considered in determining the total bacterial count (CFU/mL).

The Organic amendment (O) was the only solid sample analyzed. It was first diluted to 1 g/mL of MilliQ water (Merck, Barcelona, Spain). Then, 100 µL of this diluted sample was inoculated into the different culture media. These additional steps were also considered in determining the total bacterial count (CFU/mL).

### 3.4. Culture Media and Bacterial Isolation

Samples were inoculated into ten different culture media to grow bacteria with clinically relevant resistance phenotypes.

**Figure 11:**
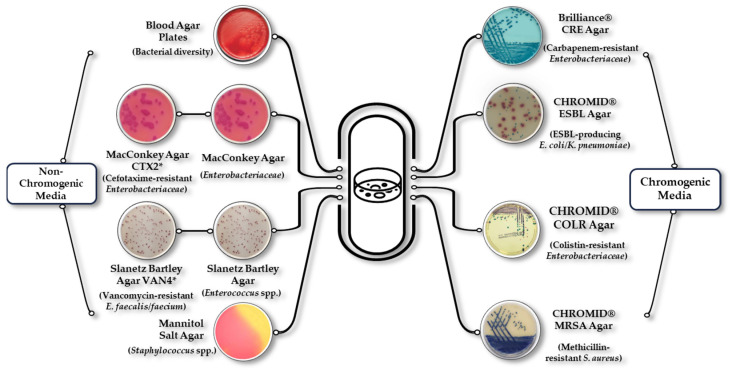
Culture media used for the isolation of bacteria with relevant resistance phenotypes. * In the case of MacConkey Agar, plates with and without cefotaxime (2 µg/mL) were used. In the case of Slanetz–Bartley Agar, plates with and without vancomycin (4 µg/mL) were used.

Commercial and chromogenic selective media supplemented with antibiotics were used: Brilliance^®^ CRE Agar (Oxoid, Madrid, Spain) for the isolation of Carbapenem-resistant *Enterobacteriaceae* (CR-E); CHROMID^®^ ESBL Agar (BioMérieux, Madrid, Spain) for Extended-Spectrum β-Lactamase-producing *E. coli*/*Klebsiella pneumoniae* (ESBL-Ec/Kp); CHROMID^®^ COLR Agar (BioMérieux, Madrid, Spain) for Colistin-resistant *Enterobacteriaceae* (COL^R^-E); and CHROMID^®^ MRSA Agar (BioMérieux, Madrid, Spain) for Methicillin-resistant *Staphylococcus aureus* (MRSA) (Figure 11).

Simultaneous inoculation was performed in non-chromogenic and specific media with/without antibiotics: Blood Agar (BA) plates (Oxoid, Madrid, Spain) for bacterial diversity; MacConkey Agar (Condalab, Madrid, Spain) non-supplemented and supplemented with 2 µg/mL of cefotaxime for the isolation of *Enterobacteriaceae* and CTX^R^-*Enterobacteriaceae*/ESBL-Ec/Kp, respectively; Mannitol Salt Agar (Condalab, Madrid, Spain) for the isolation of *Staphylococcus* spp.; and Slanetz–Bartley (SB) Agar (Condalab, Madrid, Spain) non-supplemented and supplemented with 4 µg/mL of vancomycin for the isolation of enterococci and Vancomycin-resistant *Enterococcus faecium*/*faecalis* (VR-*E. faecium*/*faecalis*), respectively. In this way, the ratio of resistant bacteria to the total number of bacteria was determined for each of the microorganisms tested (Figure 11).

After inoculation, all the plates were incubated for 24–48 h at 37 °C; after this time, 2 or 3 colonies per sample and organism type/resistance phenotype were recovered and further studied and characterized.

### 3.5. Identification and Preservation of Bacterial Isolates

Matrix-assisted laser desorption/ionization time of flight mass spectrometry (MALDI-TOF) was used to identify isolates. For this assay, the standard protein extraction protocol recommended by Bruker (Daltonics, Bremen, Germany) was followed (MALDI-TOF Biotyper^®^, Bruker). Isolates with clinically relevant resistance phenotypes or microorganisms of genus or species of interest were preserved in Difco™ Skim Milk (Becton, Pont-de-Claix, France) at −80 °C.

### 3.6. Confirmation of Relevant Resistance Mechanisms

The Extended-Spectrum β-Lactamase production by the *E. coli* and *K. pneumoniae* isolates recovered from CHROMID^®^ ESBL Agar plates was tested by the double-disk diffusion method, determining the susceptibility to cefotaxime (CTX) and ceftazidime (CAZ) surrounding the disk of amoxicillin-clavulanic acid (AMC). The enhancement of the inhibition halo for CTX or CAZ in the proximity of the AMC disk is indicative of ESBL production.

In addition, the Enterobacterales recovered from the Brillance^®^ CRE Agar plates were tested for imipenem susceptibility on Mueller–Hinton Agar plates to verify imipenem resistance (IPM^R^). Moreover, the enterococci that grew in the SB Agar plates supplemented with vancomycin were tested for vancomycin resistance using vancomycin disks (EUCAST 2023, Basel, Switzerland). Finally, the isolates identified as MRSA were tested for cefoxitin resistance (predictor of Methicillin resistance) by the use of a cefoxitin disk (EUCAST 2023, Basel, Switzerland).

## 4. Discussion

Research on bacterial diversity in wastewater using a culturomics-based analysis is currently very limited, and less attention has been paid to sewage sludge in this context. This approach is of great importance and indispensable, especially when assessing the risks and real impact on public health associated with the presence of antibiotic-resistant culturable bacteria of particular relevance in the human clinical settings. The main objective of our work was the recovery of bacteria with specific phenotypes of interest, which was conditioned by the use of selective media. This work was part of a larger study in which further genotypic characterizations (PCR and qPCR assays)/sequencing of isolates with clinically relevant resistance phenotypes will be performed.

We recognize that the methodology and culture media used were designed to recover clinically relevant ARB, which introduced a bias and conditioned to some extent the detection of some genera. During this study, a collection of 914 culturable bacterial isolates (31 genera and 90 species) was recovered from all types of samples analyzed. The effluent samples showed the highest number of different genera and species, followed by the influent samples, with *Escherichia*, *Aeromonas*, *Staphylococcus*, and *Klebsiella* being the most abundant bacteria in both cases. However, an overall decrease in bacterial diversity was observed in the amendment sludge samples (including digested sludge and organic amendment). *Escherichia* remained the predominant genus, followed by *Aeromonas*, and although there was a decrease in the percentage of *Staphylococcus* in the organic amendment compared to the influent, there was also an increase in the percentage of the genera *Klebsiella* and *Enterococcus*.

It is noteworthy that our results showed that the treatment processes in the analyzed WWTPs are not entirely effective in reducing bacterial diversity, as significant numbers of different culturable genera and species were still present in the effluent and sludge samples. This finding is not commonly reported in other studies which often focus on the reduction of bacterial load rather than diversity.

Due to the differences in the methodologies implemented (both in the WWTPs treatment and also for microbiological analysis), it would not be appropriate to compare our results in detail with those of other studies, although general comparisons are possible. For instance, a high bacterial diversity was also reported in untreated wastewater from Chinese hospitals, with *Escherichia* (34–74%) being the predominant genera detected, followed by *Acinetobacter* spp. (4–16%) [25]. However, *Acinetobacter* was not a predominant genus in our study. *Enterobacteriaceae* and *Aeromonadaceae* were previously recovered from influent and effluent [26,27,28,29,30,31,32], with detection percentages very similar to those found in our study.

In the two analyzed WWTPs, a reduction of 1 to 2 log units in the *Enterobacteriaceae* abundance was observed during the wastewater and sludge treatments. These results are similar to those reported in previous studies [33].

In our study, ESBL-Ec/Kp were detected in both influent and effluent samples from the WWTPs analyzed. Our findings indicate a reduction of 1–3 log units in ESBL-Ec/Kp abundance during the wastewater treatment process. These results were consistent with previous studies that have shown wastewater treatment reduces the frequency of ESBL-Ec in the effluent but not to an extent that ensures complete elimination.

Extended-Spectrum β-Lactamase-producing *Enterobacteriaceae* have been widely described in WWTPs from different countries and continents [18,34]. It has been previously observed that the wastewater treatment reduces the abundance of ESBL-Ec in the effluent, but also increases their proportion in relation to the total number of *E. coli* [35,36].

The persistence of ESBL-producing bacteria in treated wastewater is of concern, because it suggests that these bacteria can be discharged into surface waters, potentially spreading antimicrobial resistance downstream. The two analyzed WWTPs were confirmed hotspots for the spread of ESBL-Ec/Kp to surface waters. This finding was in line with other reports that identified WWTPs as hotspots for the dissemination of ARB into the environment [18,20,34].

Our study also showed that although there was a decrease in the abundance of ESBL-Ec/Kp during sludge treatment, these ARB were still detected in the amendment sludge samples from WWTP-1, while in WWTP-2, ESBL-Ec were detected in the digested sludge, but not in the organic amendment samples. This underscores the importance of monitoring sewage sludge as a major source of ARB whose persistent contamination poses a risk for reuse in agriculture, an issue that has been less well-documented in other studies [37].

Carbapenem-resistant *Enterobacteriaceae* (CR-E) are among the most concerning ARB and pose a significant threat to public health, especially in hospital settings, and their presence in the environment exacerbates the problem. Our results indicated a reduction of 1–3 log units in the abundance of CR-E in the effluent during wastewater treatment in WWTP-1. These results confirmed that this WWTP is a hotspot for the spread of CR-E to surface waters and are consistent with other studies reporting partial reduction but not complete elimination of CR-E through conventional wastewater treatment processes [23,38,39].

Wastewater treatment plants have been identified as important sources of clinically relevant CR-E in several countries, including Brazil, Portugal, Lebanon, Switzerland, Poland, Croatia, the Netherlands, Spain, and the United States [23,24,26,32,38,40,41,42,43].

In contrast, only IPM^R^-*C. freundii* was detected in the effluent samples of WWTP-2, which, unlike WWTP-1, does not have a pasteurization process prior to sludge digestion.

The persistence of CR-E in sludge samples is especially problematic given that sludge is often repurposed as an organic amendment in agriculture. To our knowledge, there are few culturomics-based studies demonstrating the presence of CR-E in sewage sludge samples [37,44]. CR-E have been previously recovered from sewage sludge samples but only in the early stage of treatment [44].

During sludge treatment in WWTP-1, the abundance of CR-E was practically constant in the organic amendment samples. These results align with findings reported in a previous study [40], where the authors indicated that anaerobic digestion increased the abundance of CR-E.

IPM^R^-*R. ornithinolytica* was recovered in the early stages of the sludge treatment from WWTP-1. This is a relatively rare finding as this emerging pathogen has not been well-studied in environmental samples. *Raoultella* spp. (formerly *Klebsiella* spp.) is mostly recovered from aquatic environments and soils, so detection of this ARB in clinical settings is uncommon, and therefore it has not been well studied compared to other microorganisms [45]. IPM^R^-*R. ornithinolytica* is currently of particular concern as an emerging pathogen causing nosocomial infections associated with the urinary and gastrointestinal tracts and pneumonia cases [45,46,47,48]. The first description of IPM^R^-*R. ornithinolytica* was reported from hospital samples in Ohio in 2009 [49], and also reported in China in 2015 [50] and later again in the United States in 2019 [47].

However, environmental IPM^R^-*R. ornithinolytica* isolates sharing the same carbapenem-resistant mechanism as other clinical *R. ornithinolytica* were first reported from a Chinese WWTP in 2020 [48], also described later in 2022 [51]. This demonstrates the potential of the emerging pathogen IPM^R^-*R. ornithinolytica* to spread AMR and the need for urgent action regarding the discharge and disposal of sewage sludge.

It should be underlined that CR-E were not detected in any of the sludge samples from WWTP-2. This could be due to the fact that both WWTPs have a sludge stabilization process with slight differences in the stages that could prevent the proliferation of resistant bacteria. In addition, WWTP-2 treats a smaller water volume compared to WWTP-1.

There are scarcer studies focused on the presence of Gram-positive bacteria in sewage compared to Gram-negative pathogens, and even fewer studies regarding the presence and spread of MRSA in the environment. Wastewater treatment may help to decrease MRSA levels. However, the potential of wastewater as a vehicle for the spread of MRSA to surface water has been previously demonstrated [11,14,52,53,54].

In our study, curiously, we recovered MRSA, but only in low abundance, from the effluent sample of the WWTP-1. This low abundance contrasts with other studies that report higher levels of MRSA in wastewater [4,11,16,53,55]. The detection of MRSA in the early stages of treatment from a municipal WWTP in Sweden was reported in 2009 using culturomics-based and qPCR analyses [55]. Remarkable reductions in MRSA-positive samples after treatment in four WWTPs were reported from the United States in 2012 [56]. However, MRSA has also been recovered in both raw and treated sewage also in the United States [52], and from untreated hospital effluent in Australia and Portugal [16,53]. In this sense, some authors have reported MRSA closely genetically related to clinical isolates, but only from influent and activated sludge samples [11]. Interestingly, MRSA was not detected in the sludge samples from either WWTP-1 or WWTP-2.

*Enterococcus faecium* and *E. faecalis* are the predominant enterococcal species associated with WWTPs [57]. These results are consistent with our study, where *E. faecium* and *E. faecalis* were the major species recovered. Although *E. faecalis* is most commonly reported in human feces, it appears that *E. faecium* is better adapted to sludge conditions [37].

In our study, VR-*E. faecium* was recovered in the non-treated sludge samples and even in the digested sludge samples from WWTP-1 at a high abundance (1.3 × 10^1^ CFU/mL-1 × 10^3^ CFU/mL). The first confirmed report of *van*A-mediated vancomycin resistance in enterococci (*E. faecium* and *E. durans*) was made in 1994 [58]. However, the detection of VR-*E. faecium* in sludge samples from Spanish WWTPs has been very scarcely reported.

The isolation of VRE after anaerobic digestion process was also described in Swedish WWTPs [59], demonstrating the ability of VRE to resist anaerobic digestion. In this sense, several authors have noted an increase in the frequency of VRE strains in the final steps of treatment [14,60].

A total of 101 culturable bacterial isolates were recovered from the WWTP-2 collectors [Hospital (32), Poultry slaughterhouse (36), and General slaughterhouse (33)]. The proportion of *Escherichia* spp. isolates was practically constant in the three collectors. *Aeromonas* spp. and *Klebsiella* spp. were detected at low levels in the General slaughterhouse effluent, although the highest proportion of *Staphylococcus* spp. isolates was detected in this collector. *Enterococcus* spp., *Citrobacter* spp., and *Pseudomonas* spp. were detected only in the hospital effluent samples.

Several studies have identified hospital effluent as an important source of ARB [28,61], as well as slaughterhouses, which are also considered critical points for spreading AMR [19,54,62,63]. Although, ESBL-*K. pneumoniae* or CR-E were not detected in the General slaughterhouse effluent in our study.

A higher prevalence of CR-E in hospital wastewater compared to municipal wastewater has been reported [28]. Furthermore, a high abundance of CR-*K. pneumoniae* and other CR-E was also described from a tertiary hospital in China [43]. These results are similar to ours, since among the three collectors analyzed, the Hospital effluent samples showed the highest abundance of CR-E. However, there are still different criteria for the treatment of hospital effluent prior to discharge to WWTPs, as there are authors who state that they should undergo primary treatment and others who indicate that this treatment would favor ARB, although the data are still inconclusive [25,28,35].

ESKAPE bacteria, including ESBL-*E. coli*, have been reported in Poultry slaughterhouse effluent samples in Germany [63]; while ESBL-*K. pneumoniae* have also been recovered from pigs and Poultry slaughterhouse effluent also in Germany [19]. These reports highlight the urgent need to take actions to minimize ARB discharged from hospitals and slaughterhouses.

Finally, a selected collection of bacterial isolates, including isolates with clinically relevant resistance phenotypes, was also preserved from the total culturable bacterial diversity detected in the two WWTPs and collectors during the sampling period: ESBL-*E. coli*/*K. pneumoniae*, CR-E, *Staphylococcus* spp., and *Enterococcus* spp., among other species of interest, which will be further characterized.

We recognize that the limitation of our study was the lack of Metagenomic-based analyses to complement the thorough culture-based study performed.

In the last decade, due to the significant development of molecular techniques, studies of bacterial diversity in complex ecosystems such as wastewater and sludge have primarily relied on the use of culture-independent methods. They provide a more complete analysis of the bacterial diversity present at the phylum, class, and genus levels due to their high accuracy and efficiency. Researchers believe that both approaches, with their advantages and limitations, are not mutually exclusive but complementary [64].

## 5. Conclusions

This study contributes to the growing body of evidence indicating that WWTPs play a significant role in the environmental dissemination of AMR. Bacteria with clinically relevant resistance phenotypes, such as Extended-Spectrum β-Lactamase-producing *E. coli*/*K. pneumoniae*, Carbapenem-resistant *Enterobacteriaceae*, Methicillin-resistant *S. aureus*, and Vancomycin-resistant *Enterococcus faecium* were recovered in effluent and amendment sludge samples from the two analyzed WWTPs, in some cases in high abundance.

The incomplete removal of these ARB by conventional wastewater treatment processes highlights the need for improved technologies and stricter regulations on the use of amendment sludge in agriculture. In addition, regular monitoring and surveillance of WWTPs are critical for early detection and the mitigation of risks associated with the spread of this silent pandemic.

## Data Availability

Data is contained within the article.

## Abbreviations

AMR	Antimicrobial resistance
ARB	Antimicrobial-resistant bacteria
ARGs	Antimicrobial resistance genes
COL^R^-E	Colistin-resistant *Enterobacteriaceae*
CR-E	Carbapenem-resistant *Enterobacteriaceae*
ESBL-Ec/Kp	Extended-Spectrum β-Lactamase-producing *E. coli*/*K. pneumoniae*
IPM^R^	Imipenem resistant
MRSA	Methicillin-resistant *Staphylococcus aureus*
VRE	Vancomycin-resistant *Enterococcus* spp.
VR-*E. faecium*/*faecalis*	Vancomycin-resistant *E. faecium*/*E. faecalis*
WWTPs	Wastewater treatment plants
